# Supplementary material for: Therapeutic Effect of Renifolin F on Airway Allergy in an Ovalbumin-Induced Asthma Mouse Model In Vivo
Source: Molecules. 2022 Jun 12;27(12):3789. doi: 10.3390/molecules27123789 (PMC9227769; doi:10.3390/molecules27123789)
Supplement: Supplementary file 1 [file molecules-27-03789-s001.zip › molecules-1760725-supplementary.pdf]

Supplementary Materials

Therapeutic effect of Renifolin F on airway allergy in an ovalbumin-induced asthma mouse model In vivo

Zhuya Yang [1,†], Xiaohong Li [1,†], Rongbing Fu [1,†], Min Hu [1], Yijie Wei [1], Xuhong Hu [1], Wenhong Tan [1], Xiaoyun Tong [2,*], and Feng Huang [1,*]

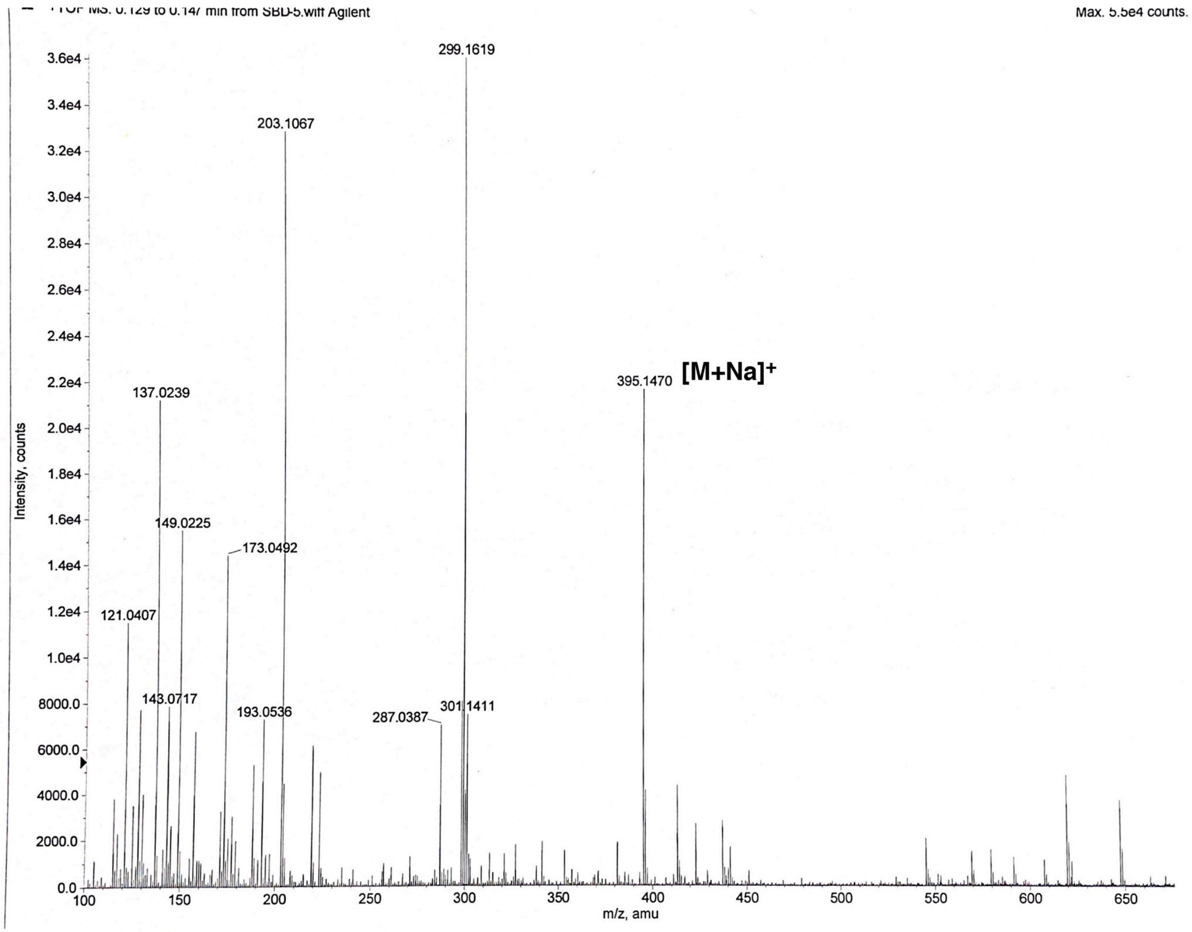

Elemental composition calculator

```
Target m/z:  +395.1470      amu
Tolerance:     +10.0000     ppm
Result type: Elemental
Max num of results: 20
Min DBE:        -0.5000      Max DBE:        +50.0000
Electron state:      OddAndEven
Num of charges:      0
Add water:  N/A
Add proton: N/A
File Name:  SBD-5.wiff
```

	Formula	Calculated m/z (amu)	mDa Error	PPM Error	DBE
1	C21 H24 O6 Na	395.1470	-0.0585	-0.1480	9.5
2	C23 H23 O6	395.1494	-2.4637	-6.2350	12.5
3	C30 H19 O	395.1435	3.4095	8.6286	21.5

Figure S1: HRMS-ES of Renifolin F

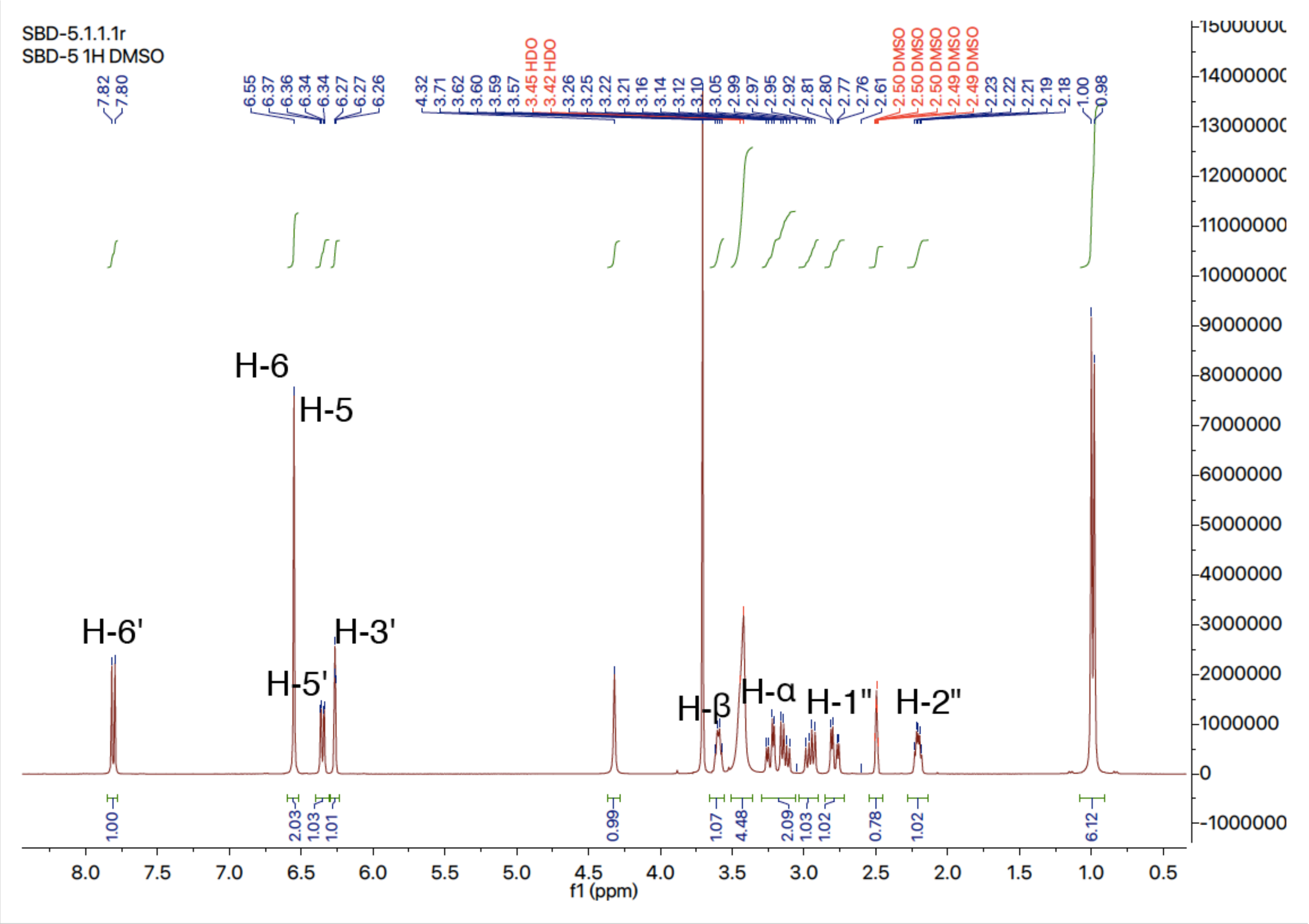

Figure S2: ^{1}H-NMR of Renifolin F (400MHz, DMSO-d_6)

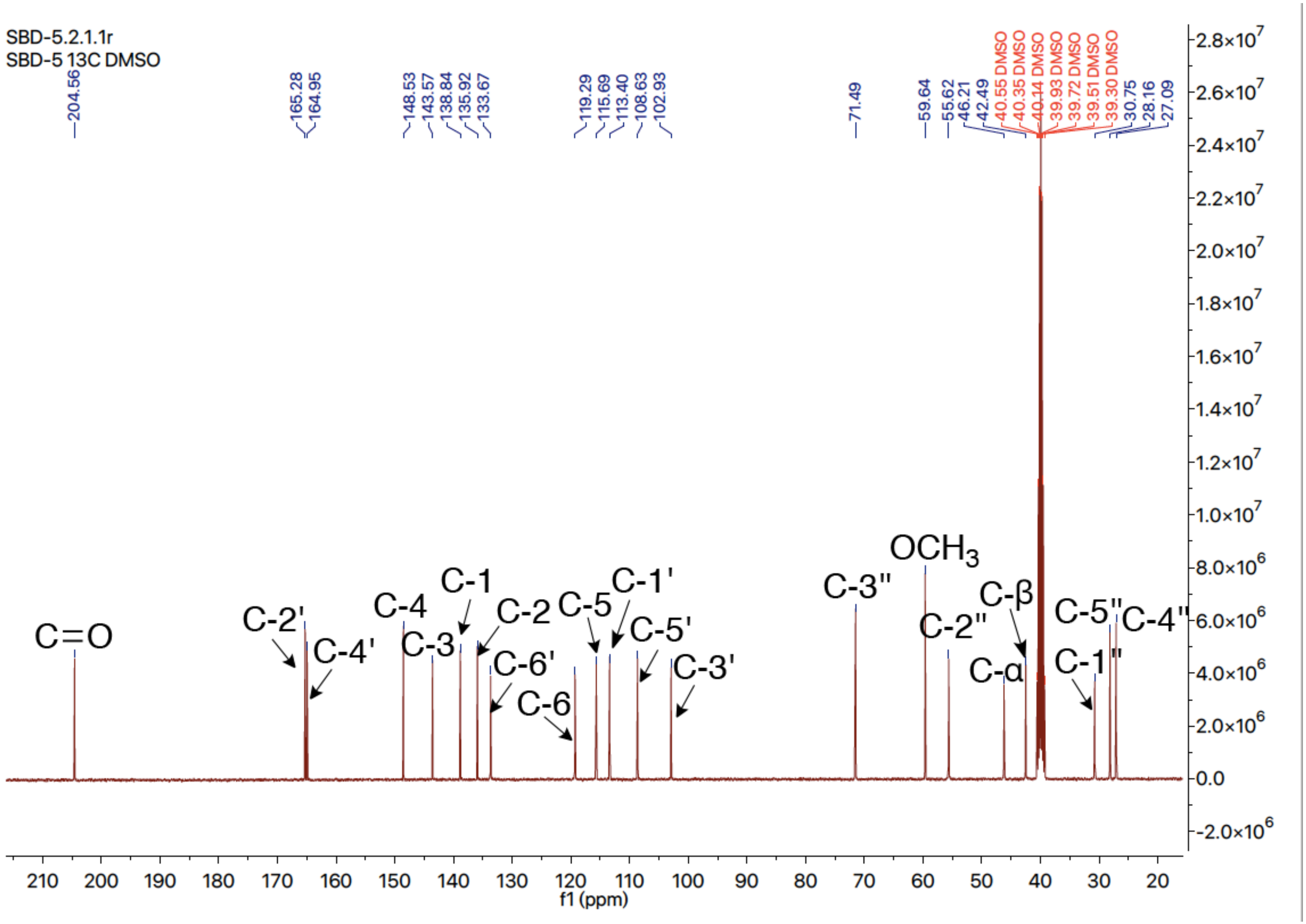

Figure S3: ^{13}C-NMR of Renifolin F (100MHz, DMSO-d_6)

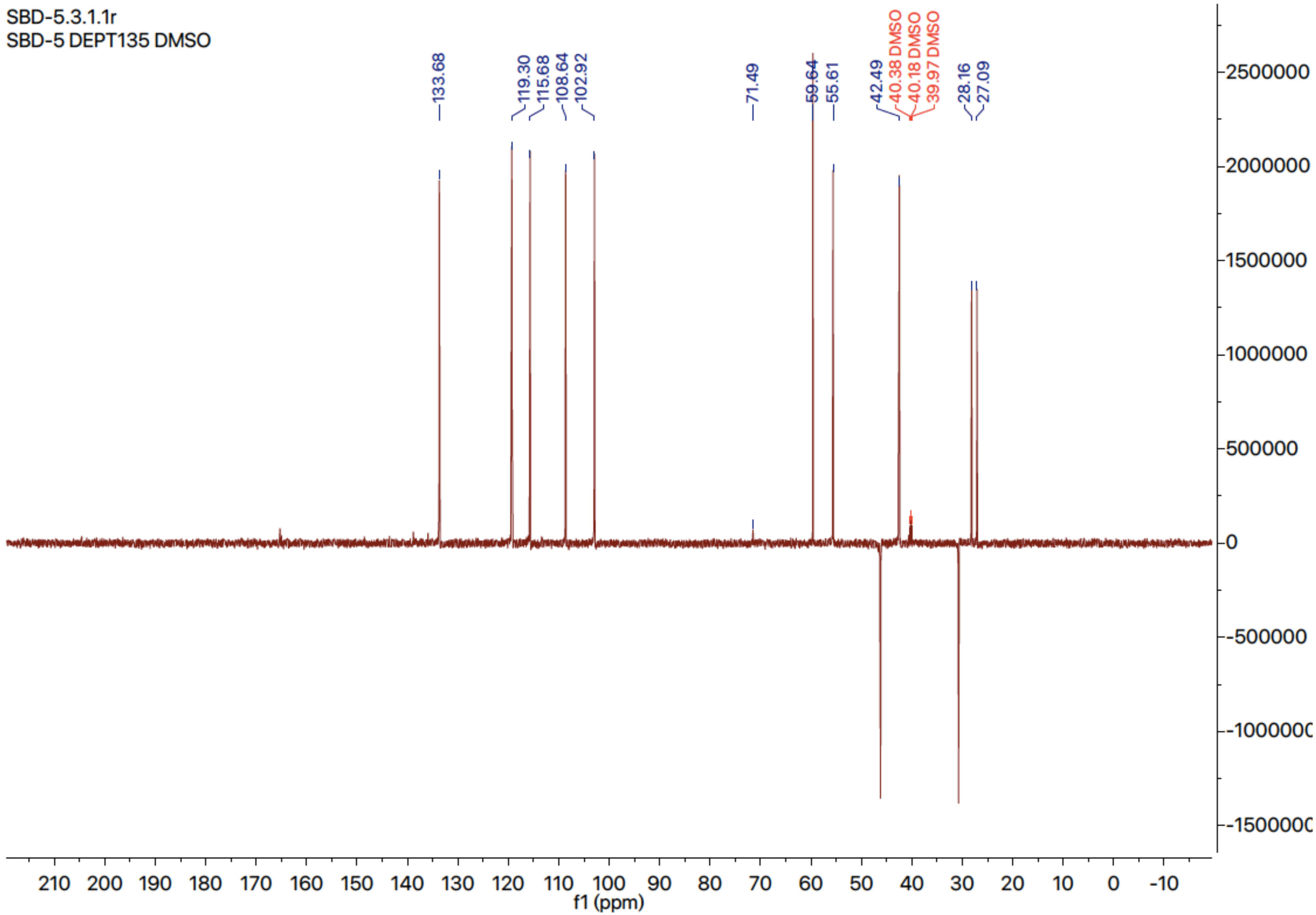

Figure S4: ^{13}C-NMR DEPT 135 of Renifolin F (100MHz, DMSO-d_6)

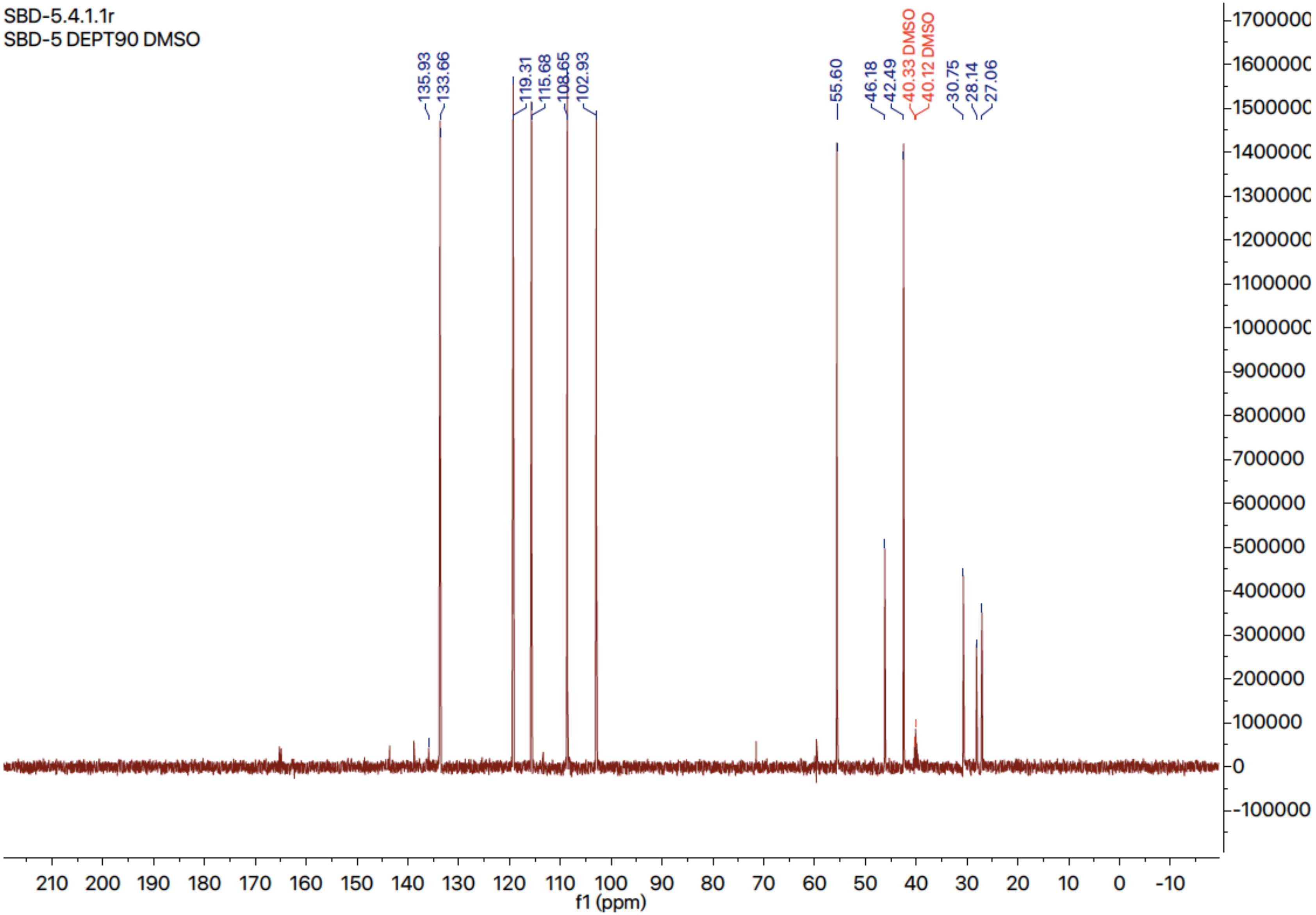

Figure S5: ^{13}C-NMR DEPT 90 of Renifolin F (100MHz, DMSO-d_6)

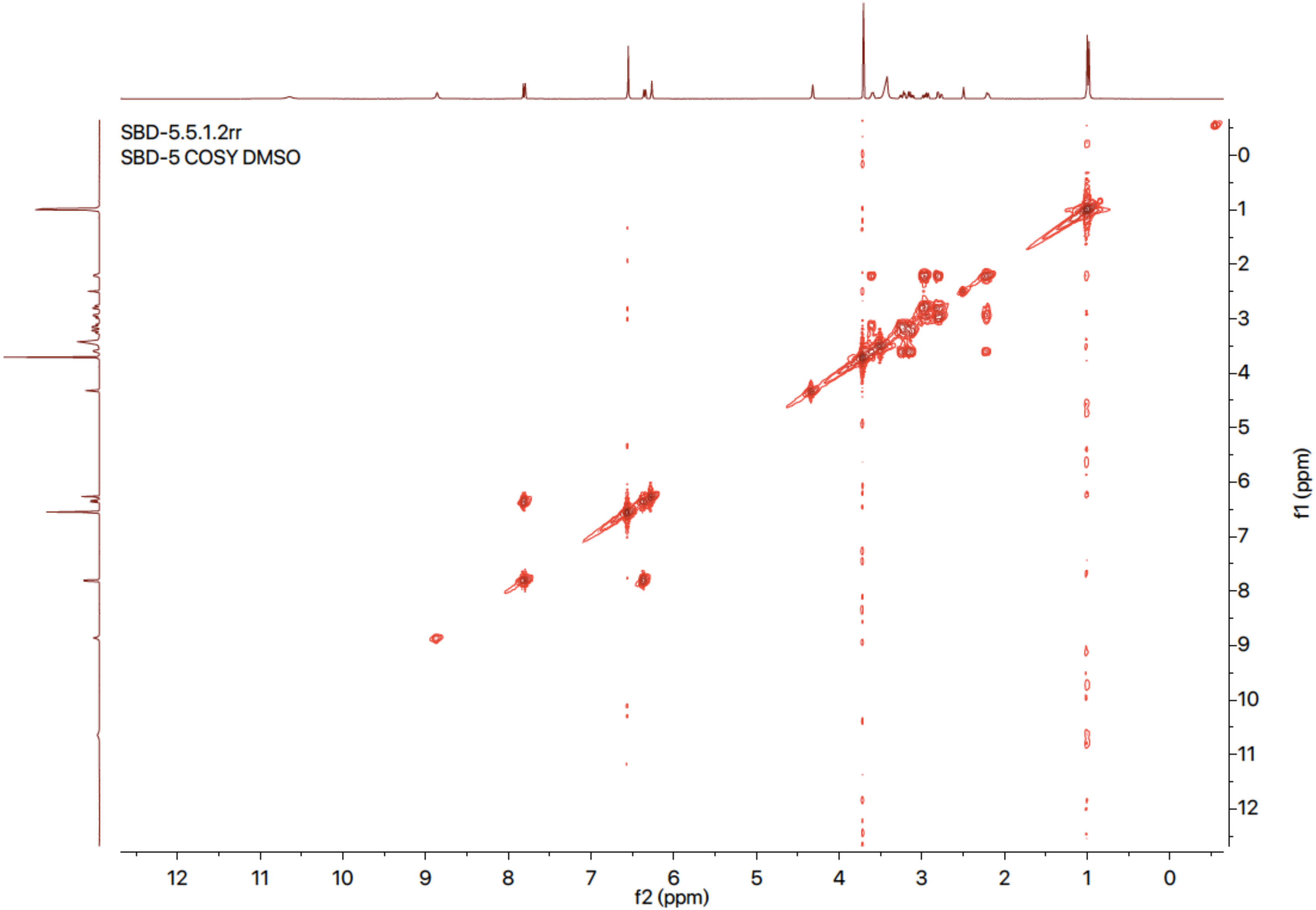

Figure S6: COSY of Renifolin F

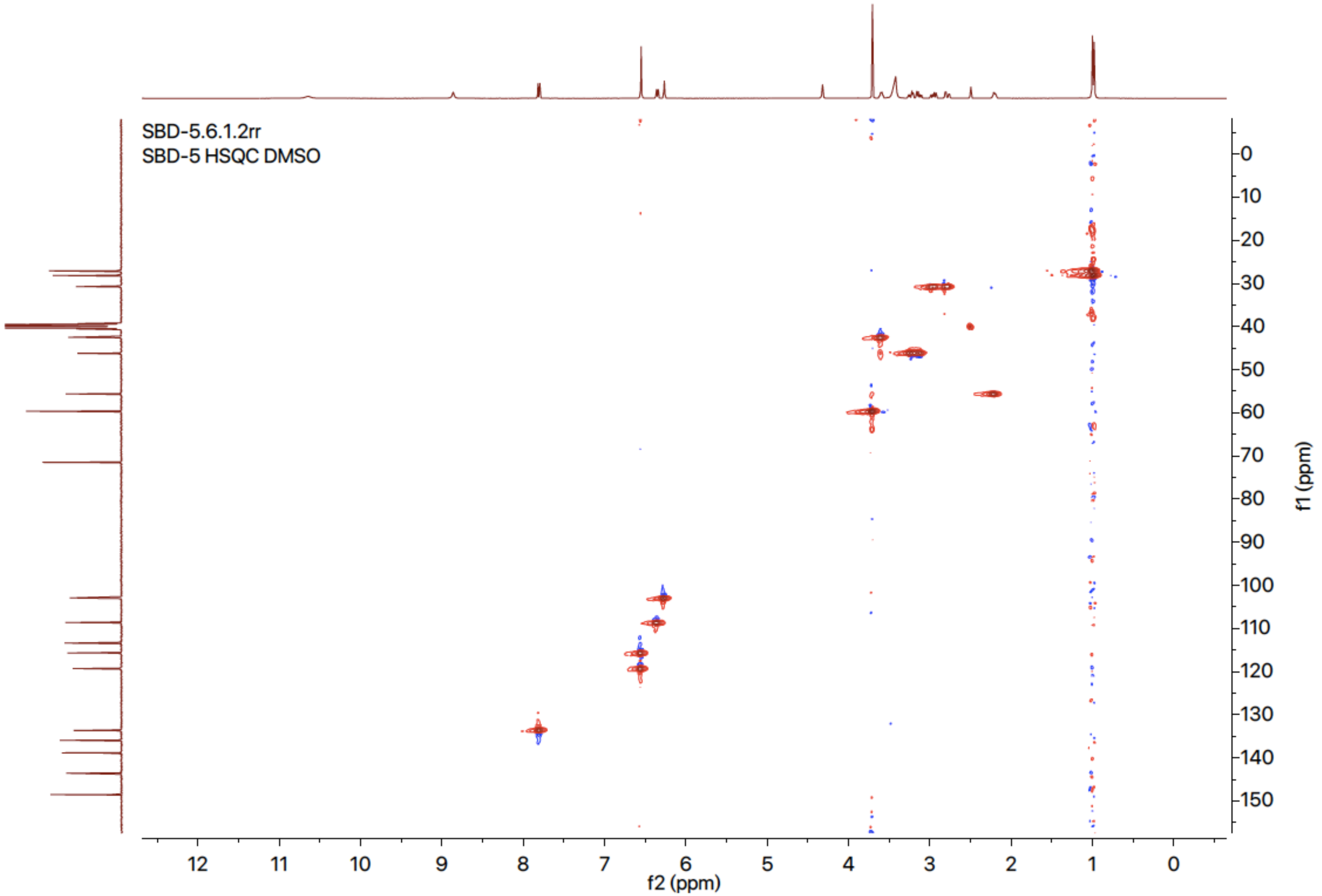

Figure S7: HSQC of Renifolin F

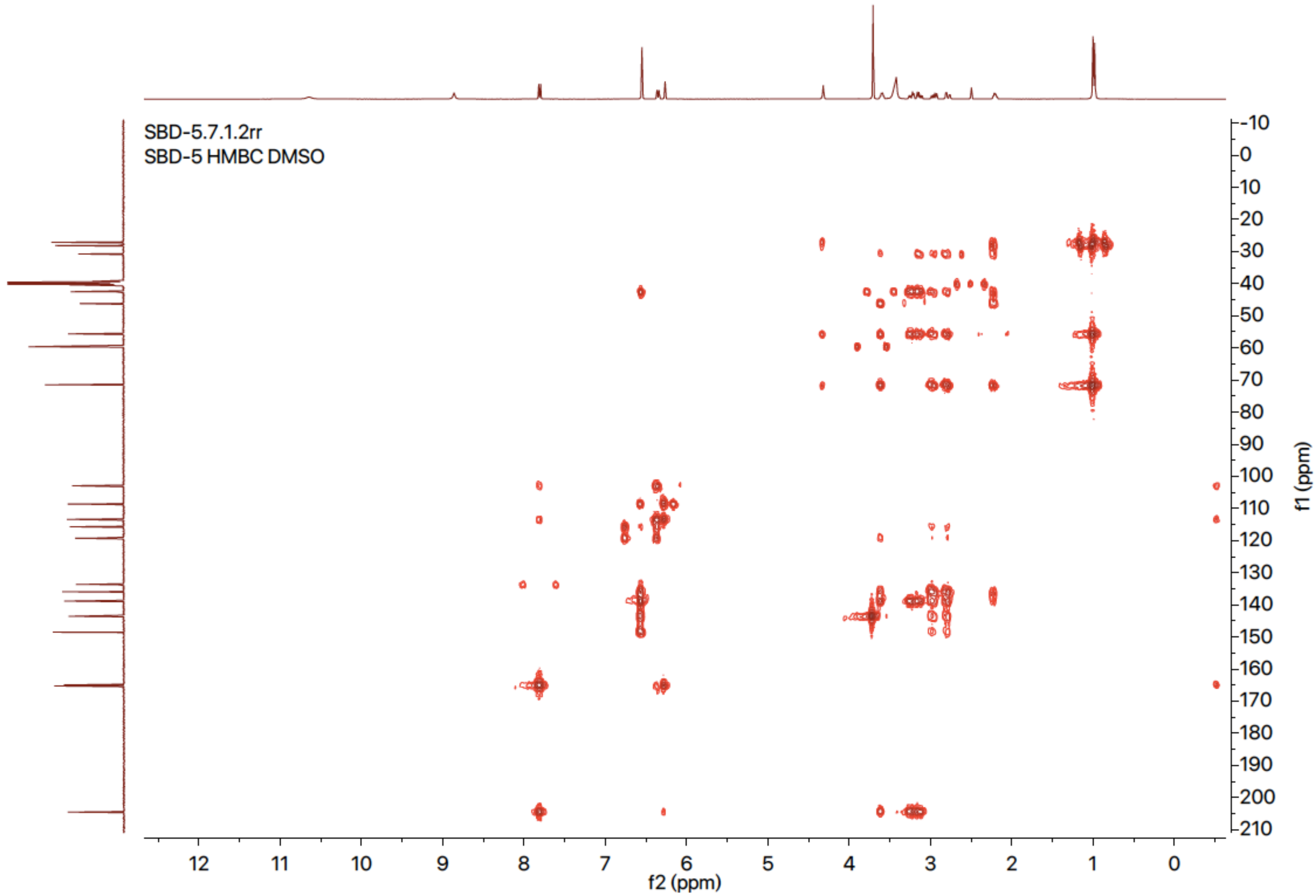

Figure S8: HMBC of Renifolin F

① Instrument

Agilent 1260 InfinityⅡ

② Solvents

Solvent A	Acetonitrile
Solvent B	0. 1% trifluoroacetic acid

③ Method

Flow(mL/min)	%A	%B
0.8	35	65

④ Column

Waters Sunfire C18 column (250 mm × 4.6 mm, 5 μm)

⑤ Absorbance

210 nm

⑥ Column temperature

26.5 ℃

⑦ Injection Volume

15 μl

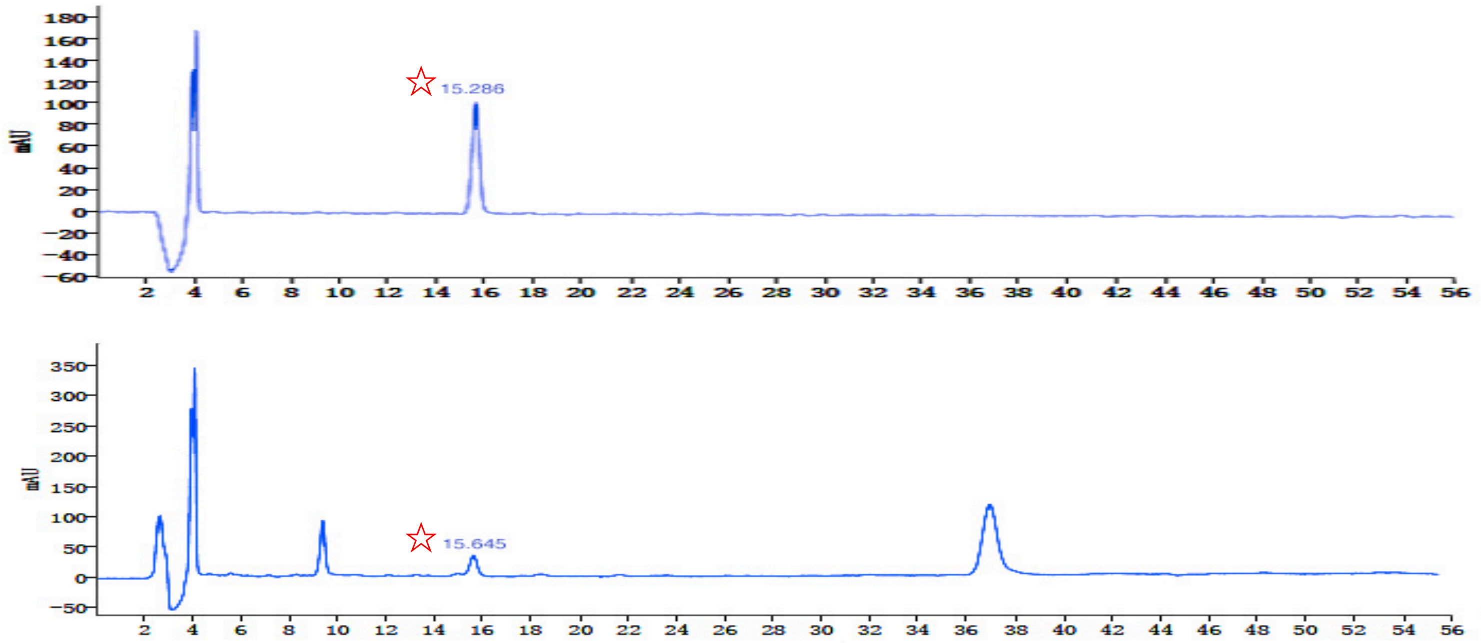

Figure S9: HPLC of Renifolin F

The upper HPLC was reference solution and the below was Tong-qian-ma-huang EtOH extract

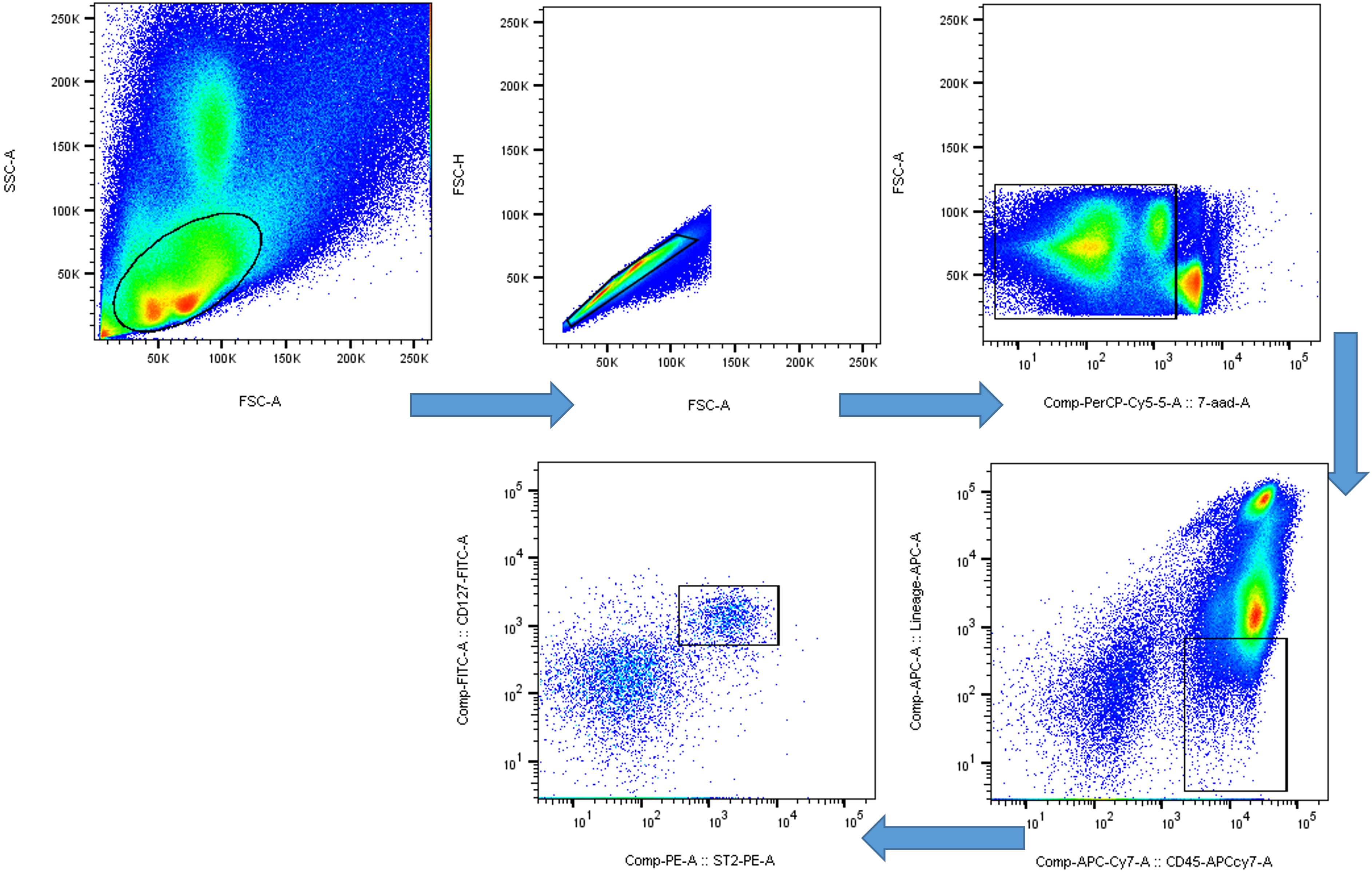

Figure S10: Flow cytometry analysis of ILC2s in lungs of OVA-induced asthma mice model
